# Japanese Encephalitis Virus Activates Autophagy as a Viral Immune Evasion Strategy

**DOI:** 10.1371/journal.pone.0052909

**Published:** 2013-01-08

**Authors:** Rui Jin, Wandi Zhu, Shengbo Cao, Rui Chen, Hui Jin, Yang Liu, Shaobo Wang, Wei Wang, Gengfu Xiao

**Affiliations:** 1 State Key Laboratory of Virology, Wuhan Institute of Virology, Chinese Academy of Sciences, Wuhan, Hubei, China; 2 State Key Laboratory of Agricultural Microbiology, Huazhong Agricultural University, Wuhan, Hubei, China; Temple University School of Medicine, United States of America

## Abstract

In addition to manipulating cellular homeostasis and survivability, autophagy also plays a crucial role in numerous viral infections. In this study, we discover that Japanese encephalitis virus (JEV) infection results in the accumulation of microtubule-associated protein 1 light chain 3-II (LC3-II) protein and GFP-LC3 puncta *in vitro* and an increase in autophagosomes/autolysosomes *in vivo*. The fusion between autophagosomes and lysosomes is essential for virus replication. Knockdown of autophagy-related genes reduced JEV replication *in vitro*, as indicated by viral RNA and protein levels. We also note that JEV infection in autophagy-impaired cells displayed active caspases cleavage and cell death. Moreover, we find that JEV induces higher type I interferon (IFN) activation in cells deficient in autophagy-related genes as the cells exhibited increased phosphorylation and dimerization of interferon regulatory factor 3 (IRF3) and mitochondrial antiviral signaling protein (MAVS) aggregation. Finally, we find that autophagy is indispensable for efficient JEV replication even in an IFN-defective background. Overall, our studies provide the first description of the mechanism of the autophagic innate immune signaling pathway during JEV infection.

## Introduction

Acute infection caused by Japanese encephalitis virus (JEV) evokes several distinct innate immune responses, which function partially by a molecular mechanism involving the RIG-I/IRF-3 and PI3K/NF-κB signaling pathways. Activation of the signaling network results in significant changes in the expression of multiple inflammatory cytokines, chemokines and IFN-inducible proteins [1], [2], [3], which not only perform their anti-viral functions but also contribute to JEV pathogenesis, resulting in encephalitis.

The major function of autophagy is to deliver damaged organelles and long-lived proteins to the lysosomal machinery, thereby balancing synthesis, degradation, and subsequent recycling. Especially in extreme environments, autophagy aids in the reallocation of nutrients from unnecessary processes to more essential processes to maintain cellular homeostasis [4]. Classical autophagy can be divided into three major steps. The first step involves vesicle regulation and nucleation of an isolation membrane. Second, the isolation membrane goes through elongation and fusion processes to form the autophagosome, which is a double-membrane vesicle that sequesters the cytoplasmic materials and organelles. The last major step is docking and fusion of the completed autophagosomes with lysosomes to form autolysosomes, where the captured materials are degraded [5].

Currently, the autophagy pathway has been shown to be activated by a growing number of viruses. The replication of some viruses is impaired by the autophagy pathway, whereas other viruses may utilize the pathway to facilitate their propagation. In the case of herpes simplex virus 1 (HSV-1), virus evasion of autophagy machinery is essential for viral replication and lethal encephalitis [6], but dengue virus (DENV) and hepatitis C virus (HCV) benefit from autophagy to enhance their replication [7], [8]. Li et al. reported for the first time that the cellular autophagy process is involved in JEV infection and that the inoculated viral particles traffic to autophagosomes for subsequent steps of viral infection [9]. However, the exact autophagic regulation mechanism in JEV replication is less clear. In our study, we investigated whether JEV-induced autophagy regulated the innate immunity pathway. We found that the suppression of autophagy in JEV-infected cells correlated with an enhanced innate immune response. These autophagy-dependent alterations in IFN expression are necessary for efficient JEV replication. We also found that autophagy can prolong cell survival and can postpone cell death during infection. Taken together, our studies provide the first description of the mechanism of the autophagic innate immune signaling pathway during JEV infection.

## Results

### JEV Infection Induces Autophagy

**Figure 1:**
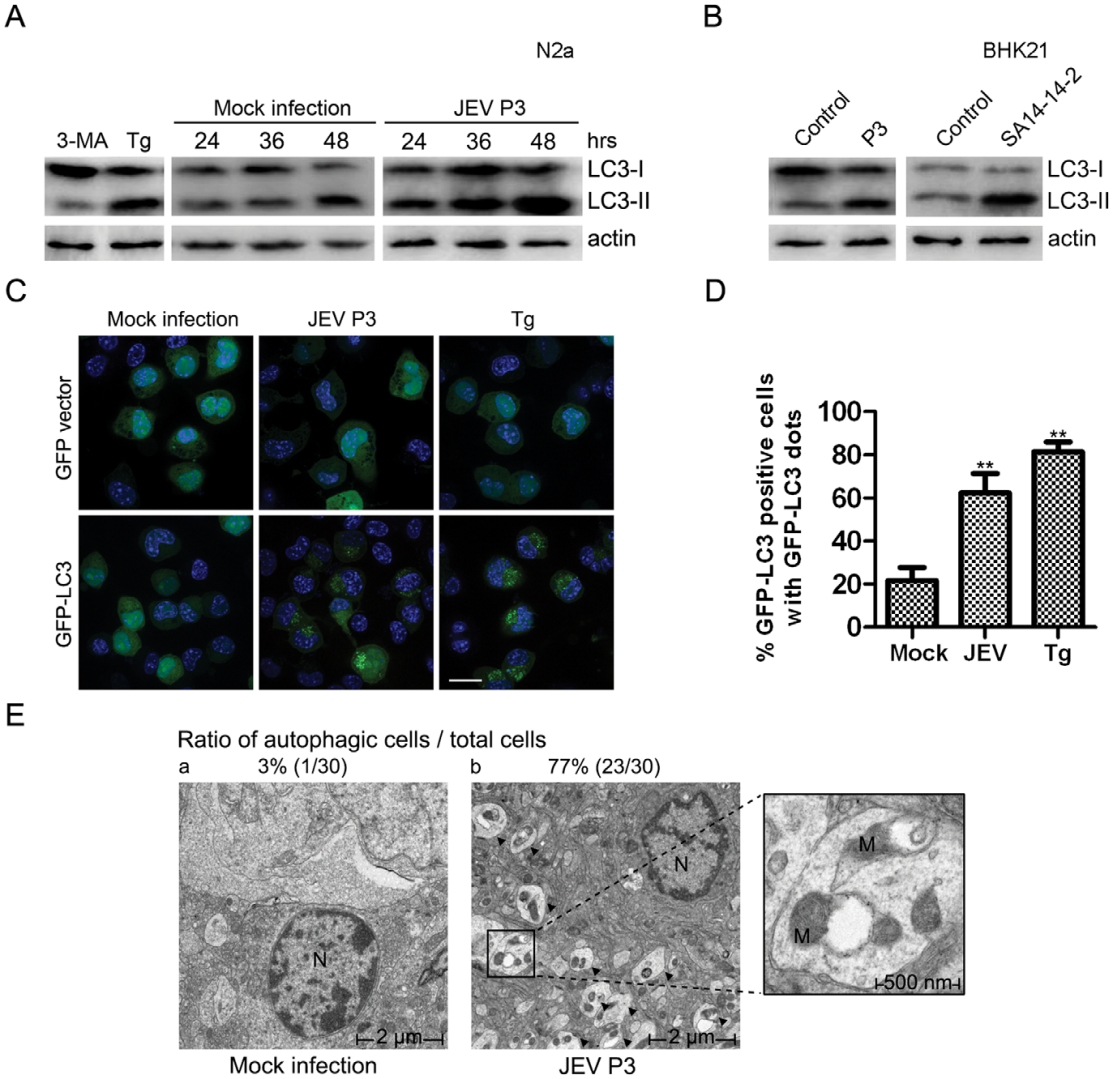
Induction of autophagy by JEV. (A) N2a cells were treated with Tg (1 ug/ml, 24 hours) or 3-MA (10 mM, 24 hours) or infected or mock-infected with P3 virus (MOI = 1) for 48 hours. Cell lysates from different time points were harvested for immunoblotting. (B) BHK21 cells were infected or mock-infected with JEV P3 or SA14-14-2 virus for 48 hours, and the cell lysates were harvested for immunoblotting. (C) Twelve hours after transfection with GFP-LC3 or the GFP vector plasmid, N2A cells were mock-infected or infected with JEV P3 (MOI = 1) virus for 48 hours or treated with Tg for 24 hours. The nuclei were stained with DAPI, and the cells were observed under a fluorescence microscope. The white scale bar is 20 µm. (D) Cells were treated as in (C); cells containing five or more GFP-LC3 dots were defined as autophagy-positive cells. The percentages of cells with characteristic punctate GFP-LC3 ﬂuorescence relative to all GFP-positive cells were calculated. The results represent the mean data from three independent experiments. The statistical significance of changes in viral RNA and virus yield compared with the control were calculated by t-test. **: *P*<0.01. (E) The ratio of autophagy (autophagic cells/total cells) was determined by counting the number of cells containing autophagic vacuoles among a total of 30 randomly selected cells. The larger boxed images on the right represent enlargements of the smaller boxed insets of (b). The arrows indicate representative autophagosome-like structures. The cell nucleus and mitochondria are abbreviated as N and M. a) mock-infected and b) JEV-infected mouse brains. The magnification is 2500 X.

Neuro2a mouse neuroblastoma cells (N2a) were employed because this cell line is permissive to JEV infection [10]. To monitor the autophagy process, we first analyzed an autophagic marker protein, microtubule-associated protein 1 light chain 3 (LC3). Monitoring of LC3 is the most widely used method to detect cellular autophagic activity. When the autophagic process takes place, the cytosolic LC3-I is cleaved and conjugated with phosphatidylethanolamine (PE), converting into the autophagic form, LC3-II [11]. The ratio of LC3-II to actin is a direct indicator of the accumulation of autophagosomes in cells [12]. An increased accumulation of LC3-II was noted when the JEV P3 strain infected N2a cells, especially in the late stage of infection (Fig. 1A). The LC3-II levels in mock-infected cells did not increase as much as those in infected cells. Treatment with the ER stress inducer thapsigargin (Tg) and the PI3K inhibitor 3-methyladenine (3-MA) [13], [14] served as positive and negative controls, respectively (Fig. 1A). Furthermore, both P3 and the attenuated SA14-14-2 strain induced autophagy in BHK21 cells, indicating a universal JEV strain effect for various cell types (Fig. 1B).

To provide further evidence of JEV-induced autophagosome formation, fluorescent plasmids and microscopy were used to detect fluorescent LC3 redistribution, which represented the autophagosomes. Cells transfected with GFP-LC3 induced evident GFP dots only when infected with JEV or following positive treatment with Tg (Fig. 1C, D).

Transmission electron microscopy (TEM) remains the gold standard for identifying autophagy formation at the ultrastructural level. Therefore, brain tissue slices of infected and non-infected naive mice were monitored using TEM. In Fig. 1E, b, large numbers of phagophores are visible. The limiting membrane is partially visible as two bilayers separated by a narrow cleft, as detailed in the close-up view, but vacuoles were rarely noted in the control group (Fig. 1E, a). These data demonstrate that JEV infection can trigger autophagy machinery and can induce autophagosome formation *in* *vitro* and *in* *vivo*.

### Autophagosome-lysosome Fusion is Essential for JEV Replication

**Figure 2:**
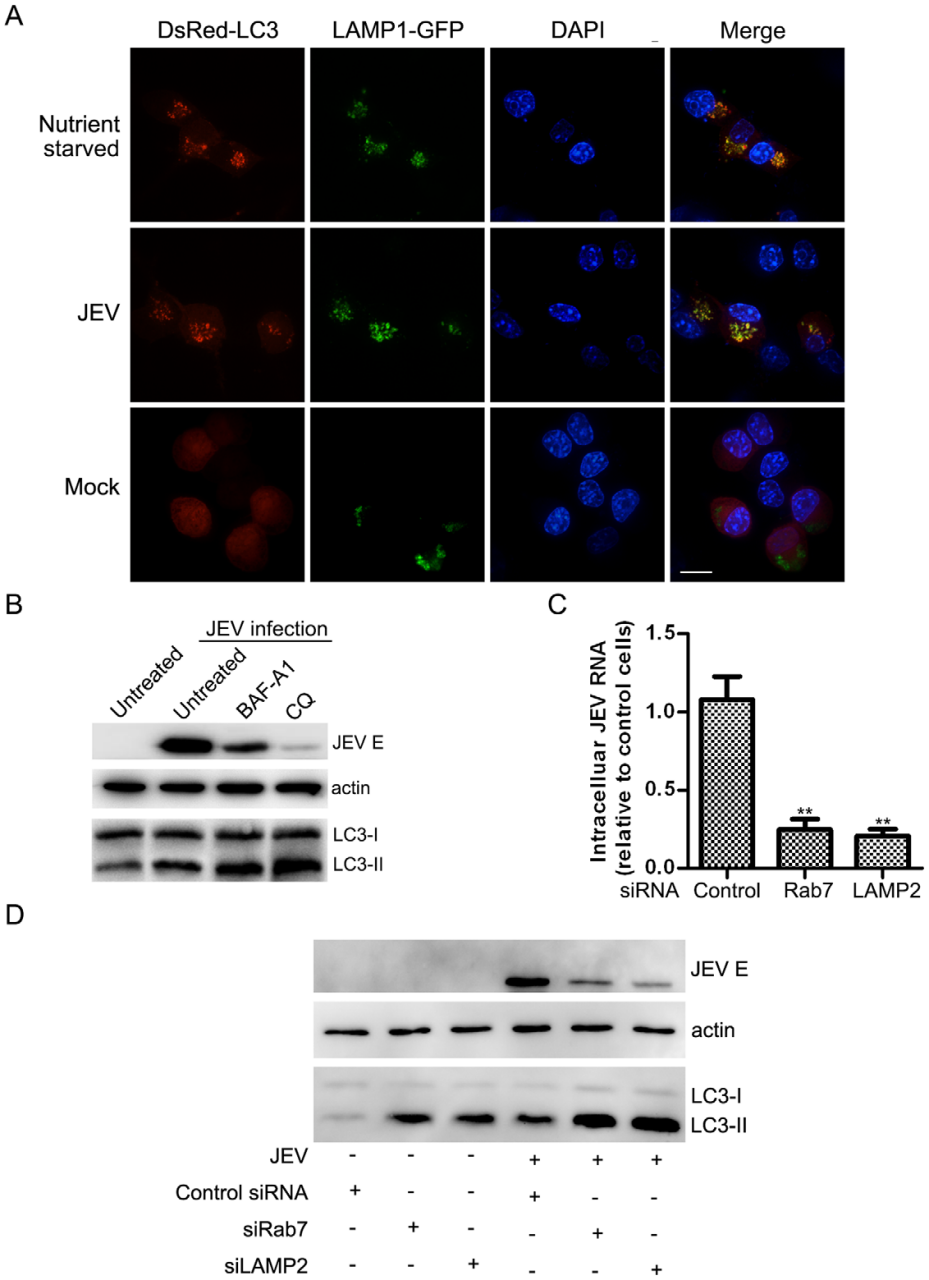
Autophagosome maturation induced by JEV infection. (A) N2a cells were co-transfected with the DsRed-LC3 and LAMP1-GFP plasmids. Twelve hours after transfection, the cells were then challenged with JEV. Thirty-six hours post-infection, the cells were fixed, and the nuclei were stained with DAPI. The cells were observed under a confocal ﬂuorescence microscope. The white scale bar is 20 µm. (B) N2a cells were mock- or JEV-infected at an MOI of 5 for 24 hours and then treated with BAF-A1 (100 nM), CQ (50 µM) or vehicle control and then lysed for analysis. N2a cells were transfected with siRNA oligonucleotides targeting the indicated gene. Twenty-four hours later, the cells were infected at an MOI of 0.1 for 72 hours and then harvested and lysed for RNA (C) and protein (D) analysis. Data for JEV RNA levels represent means with SEM of three independent experiments, compared with the control. The statistical significance of changes in viral RNA compared with the control was calculated by t-test. **: *P*<0.01.

Shuttling of autophagosomes to lysosomes for breakdown and degradation is the final step in classical autophagy. However, some viruses only induce the early stage but block autophagolysosomal fusion [15], [16]. One interesting question is whether JEV infection-induced autophagy includes vesicle fusion for breakdown and degradation. After co-transfection with two plasmids, red LC3 and green LAMP1 (lysosome-associated membrane protein 1, a lysosome marker), cells were infected, mock-infected or nutrient-starved and were then monitored via confocal microscopy. Our results indicated that most of the cells displayed a bright, punctuate red LC3 signal co-localized with green lysosomes after infection with JEV or nutrient starvation, which was not observed in the control group (Fig. 2A). Past reports indicate that autophagosome fusion with early and late endosome is needed for fusion with lysosomes [17], and we also observed that autophagy formation co-localized with the Ras-related GTPases 5 and 7 (Rab5 and Rab7, representing early and late endosome markers, respectively) (Fig. S1, 2).

To investigate the importance of autophagosome maturation, we analyzed the process with two drugs: chloroquine (CQ), which raises the lysosomal pH and ultimately inhibits the fusion between autophagosomes and lysosomes, blocking a late step in macroautophagy [18], and bafilomycin A1 (BAF-A1), an inhibitor of the vacuolar (V)-type ATPase that alters the pH and membrane potential of acidic compartments, ultimately resulting in blockage of autophagosome-lysosome fusion [18]. Although additional CQ and BAF-A1 treatment induced more LC3-II accumulation compared to the control, JEV E protein expression was concomitantly reduced (Fig. 2B). Furthermore, we performed an experiment using two siRNA oligonucleotides that targeted key proteins for fusion, Rab7 and lysosome-associated membrane protein type 2 (LAMP-2), both of which participate in the autolysosome maturation step [18]. The siRNA silencing effect was tested as shown in Fig. S3. Knocking down Rab7 or LAMP2 greatly reduced virus mRNA and protein expression (Fig. 2C, D). Collectively, these results convincingly underscore the pivotal role of the induction of autolysosome maturation and its importance for viral replication.

### Autophagy Positively Regulates JEV Replication

**Figure 3:**
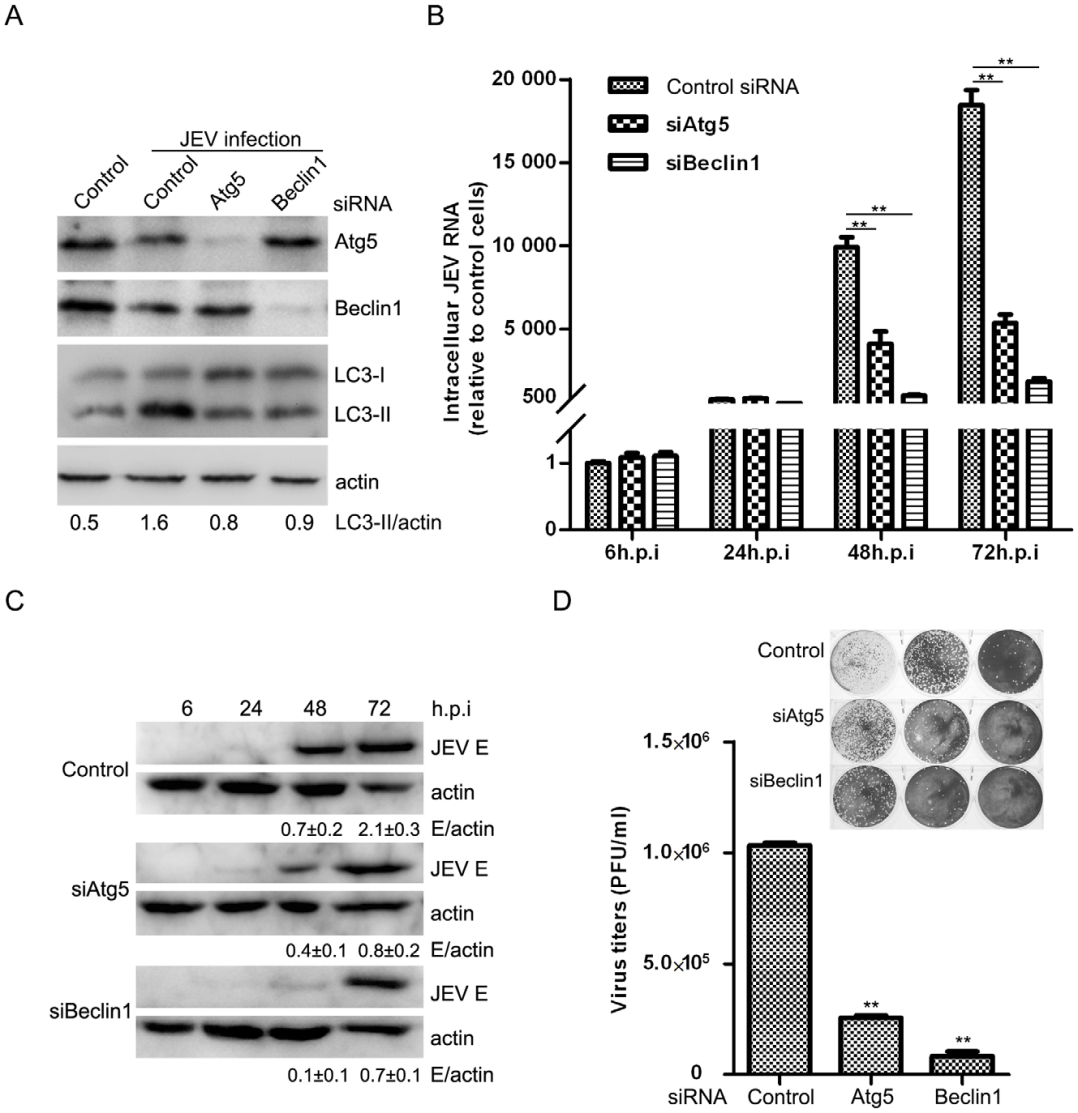
JEV Replication is dependent upon autophagy. N2a cells were transfected with siRNA oligonucleotides against Atg5 and Beclin1. Twenty-four hours later, the cells were infected at an MOI of 0.1. The cells were harvested at the indicated time for RNA (B) and immunoblotting analysis (A, C). The fold-induction ratio of JEV E/actin was quantified by densitometric analysis using FluorChemHD software. (D) The 72 h p.i. culture supernatants were collected for plaque assays on BHK-21 cells. Three independent replicates were performed, and the results are presented as the means with SEM. The statistical significance of changes in viral RNA and virus yield compared with the respective controls was calculated by t-test. **: *P*<0.01.

To investigate the possible effect of the autophagic pathway on JEV RNA replication in general, cells were transiently transfected with siRNA directed against Atg5, a constituent of the Atg12–Atg5-Atg16 complex, which contributes to LC3–PE conjugation [19]. siRNA was also directed against Beclin1, part of the class III PtdIns 3-kinase complex involved in activating macroautophagy [20]. siRNA-mediated transient knock-down of Atg5 and Beclin1 specifically inhibited the JEV-triggered accumulation of LC3-II, as shown in Fig. 3A. Cells were infected with the same MOI, and then total cellular RNA was extracted at different time points and analyzed by qRT-PCR (Fig. 3B). A significant inhibition of JEV RNA expression was observed, especially 72 hours post-infection, when greater than 70% and 90% reductions were determined in siAtg5 and siBeclin1 cells, respectively, relative to cells receiving the scrambled siRNA (Fig. 3B). Subsequently, studies at the protein level verified the reduction in JEV replication (Fig. 3C). A comparison of virus production in these two knock-downs and in control cells followed. Virus yield declined by 75% in the siAtg5 group and by 92% in the siBeclin1 group. These reductions suggested an important role for autophagic regulation in progeny virus replication (Fig. 3D). Therefore, these results suggest that autophagy facilitates JEV infection.

### Autophagy Promotes Cell Survival Under Infection Stress

**Figure 4:**
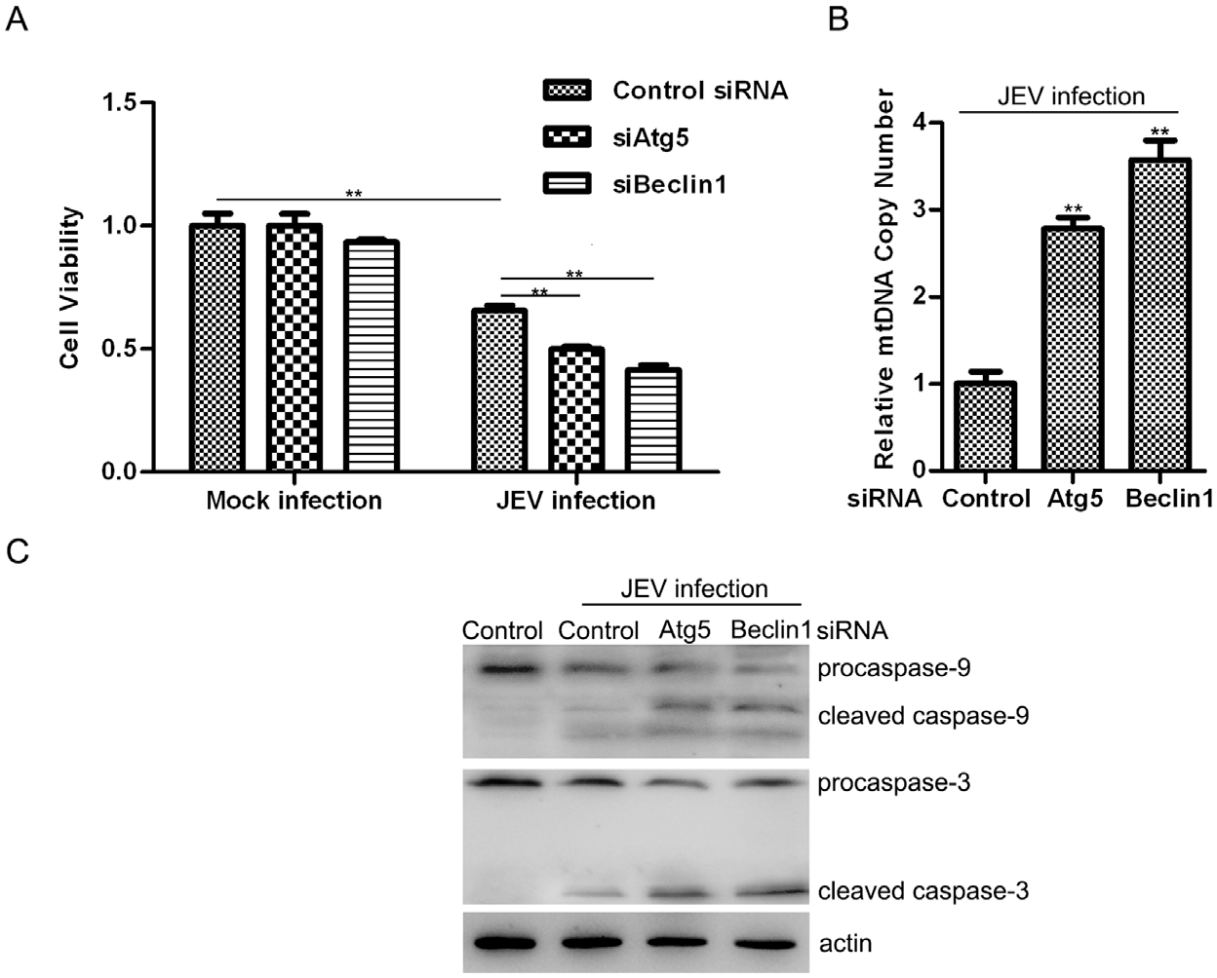
JEV infection in autophagy knock-down cells induces greater cell death. (A) Cells were treated the same as in Fig. 3B. After 72 hours post-infection, WST-8 dye (Beyotime, C0038) was add to each well, cells were incubated at 37°C for 2 h and the absorbance was determined at 450 nm using a microplate reader. N2a cells were transfected with the indicated siRNA, and 48 hours later, the cells were infected at an MOI of 1 for 48 hours. Total cellular DNA was extracted, and the mitochondrial DNA copy number was measured by quantitative PCR and normalized to nuclear DNA levels according to the ratio of mtDNA COI over 18S rDNA (B); cell proteins were also harvested, and caspase-9, caspase-3 and actin were analyzed by immunoblotting (C). Three independent replicates were performed, and the results are presented as the means with SEM. The statistical significance of changes compared with the respective controls was calculated by t-test. **: *P*<0.01.

To explore the possibility of cell survival following virus infection, we examined the viability of JEV-infected control-, siAtg5-, and siBeclin1-N2a cells. We did not observe any differences in cell viability in Atg5- or Beclin1-knock-down cells versus control uninfected N2a cells. However, cell survival diminished if autophagy was inhibited following infection (Fig. 4A). Previous research has revealed that JEV infection induces mitochondrial disruption, reactive oxygen species (ROS) production and inflammation, which are the chief reasons for apoptosis and cytopathology [21], [22]. In addition, autophagy functionally responds to damaged mitochondria cleanup by delivering unwanted proteins and organelles to lysosomes [23]. We hypothesize that autophagy-related mitochondrial dysfunction has an essential role in JEV induced cell death. We observed large numbers of double-membrane phagophores sequestered in the cytoplasmic areas in JEV-infected mouse brains (Fig. 1E), along with many vesicles containing damaged mitochondria, though few were noted in the control group. Subsequently, we quantified the relative mitochondrial DNA copy numbers as the ratio of mtDNA cytochrome c oxidase subunit I (COI) to 18S rDNA. The analysis revealed an increase (greater than two-fold) in the total numbers of mitochondria in the autophagy gene-defective groups compared with the mock group (Fig. 4B). These data imply that JEV infection might induce autophagy-mediated mitochondria damage, which could be one of the reasons for apoptosis. Caspases are a family of cysteine proteases that play essential roles in apoptosis and inflammation, and the hallmark of their activation is cleavage [24]. Fig. 4C reveals that cells exhibited more caspase-9 and caspase-3 cleavage after JEV infection and when cells suffered autophagy machinery dysfunction (Fig. 4C). In particular, enhanced apical protease caspase-9 cleavage implies a rising apoptosis rate via a mitochondrial death pathway [25]. Therefore, dysfunctional autophagy is the reason for increasing of the infected cell death.

### JEV-induced Autophagy is Negatively Correlated with Type I IFN (a Regulator of Viral Replication) Production

Previous studies have demonstrated that autophagy positively regulates JEV production and creates conditions favorable for the initiation of JEV infection, possibly by facilitating an early infection step, such as entry and uncoating [9]. However, N2a cells with comparable entry or early replication efficiencies (Fig. 3B) propagated incompatible progeny JEV, so we considered other potential roles that autophagy might play in JEV infection. Studies of vesicular stomatitis Indiana virus (VSV) and HCV have suggested that autophagy may suppress innate antiviral immunity in preparation for productive infection [26], [27], [28], so we challenged the JEV infection using IFN induction mechanisms.

**Figure 5:**
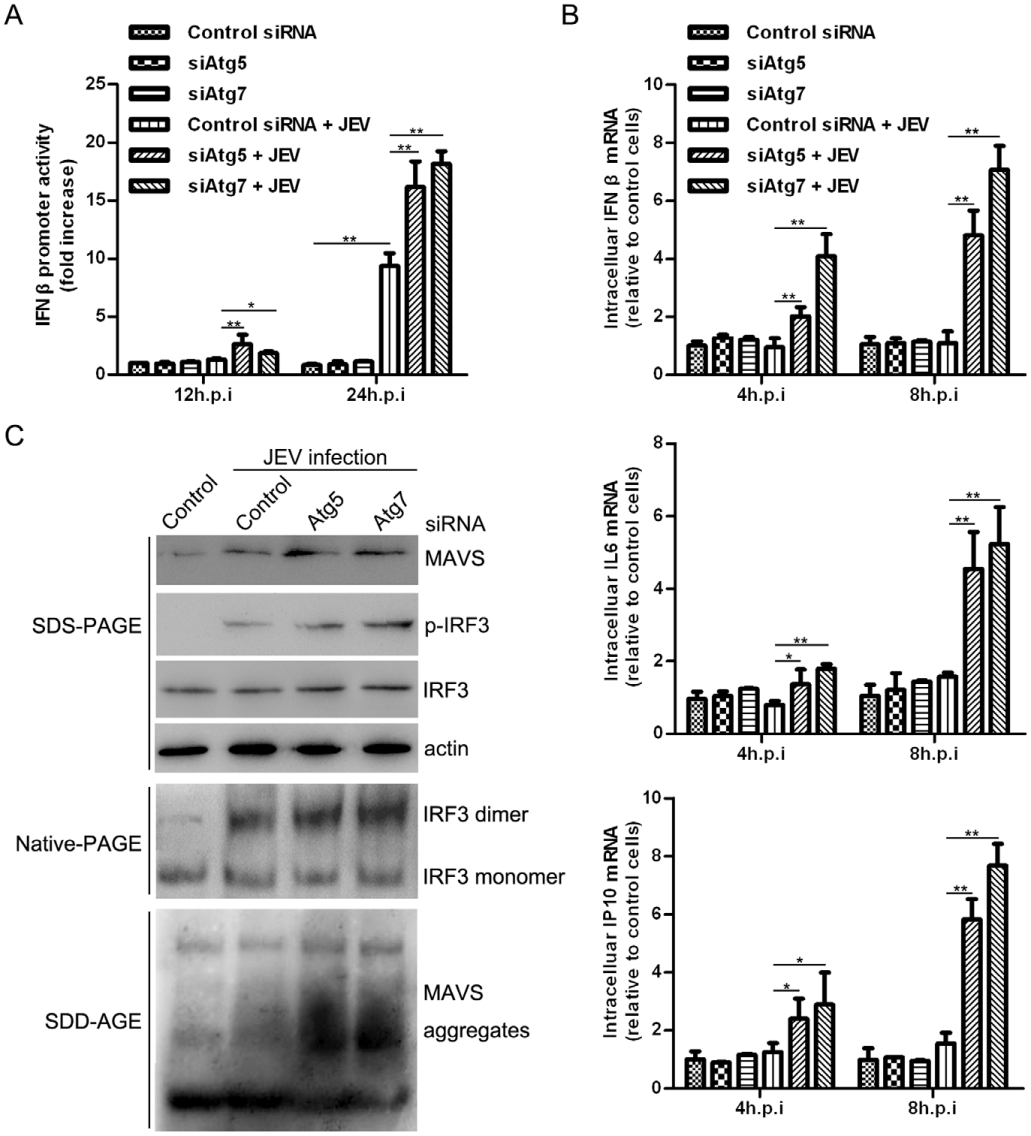
JEV infection up-regulates the IFN signaling pathway in autophagy-deficient A549 cells. (A) The cells were first transfected with the indicated siRNA, and 48 hours later the cells were then transfected with IFN β-Luc Firefly luciferase and an internal control, pRL-TK (Renilla luciferase). After 24 hours, the cells were infected with JEV at an MOI of 10. Finally, the cells were harvested at the indicated time, and dual luciferase activity was determined. (B) The cells were first transfected with the indicated siRNA, and 72 hours later the cells were infected at an MOI of 10. At different time points, total RNA was extracted for analysis. (C) The experiment was processed in parallel with (B), except that proteins used for immunoblotting were harvested at 24 hours post-infection. Three replicates were performed, and the results are presented as the means with SEM. The statistical significance of variations compared with the respective controls was calculated by t-test. *: *P*<0.05, **: *P*<0.01.

To obtain optimal results, we used an IFN-sensitive cell line, A549, which has been extensively used for IFN studies [2]. To do so, siRNA was directed against human Atg5 and Atg7 (Atg7 is an ubiquitin-activating (E1) enzyme homologue that activates both tg8/LC3 and Atg12 in an ATP-dependent process [18]) to investigate the possible effect of autophagy on the innate immunity pathway. The siRNA knock-down was tested, as shown in Fig. S4. We first performed a dual luciferase reporter assay to monitor the transcriptional regulation of the IFN β promoter. Our results implied that promoter activity was enhanced as the infection proceeded, and disruption of Atg5 and Atg7 function further accelerated the activity. The effect can be observed as early as 12 hours post-infection, and it is remarkable at 24 hours (Fig. 5A). In addition, we analyzed the mRNA levels of IFN β, IL6 and IP10 at an earlier time point. Interestingly, although IFN β, IL6 and IP10 mRNA production are almost equivalent at 8 hours post-infection when comparing infected and non-infected cells, the levels were significantly higher in Atg5 and Atg7 knock-down cells relative to control cells (Fig. 5B). There did not reveal remarkable differences in promoter activity and mRNA production in Atg5- or Atg7-knock-down cells versus control in uninfected A549 cells. We also determined the activation of interferon regulatory factor 3 (IRF3), which is a marker of the virus-induced type I IFN signaling pathway. Knock-down of Atg5 and Atg7 increased JEV-induced IRF3 phosphorylation and dimerization (Fig. 5C), which are hallmarks of IRF3 activation [29], [30]. In addition, greater accumulation levels of prion-like aggregates of mitochondrial antiviral signaling protein (MAVS), which serves as a potent activator of IRF3 [31], were detected in autophagy-deficient cells (Fig. 5C). These results suggest that the autophagic machinery influences type I IFN production.

### Autophagy is Indispensable for JEV Replication in the IFN Defective Background

**Figure 6:**
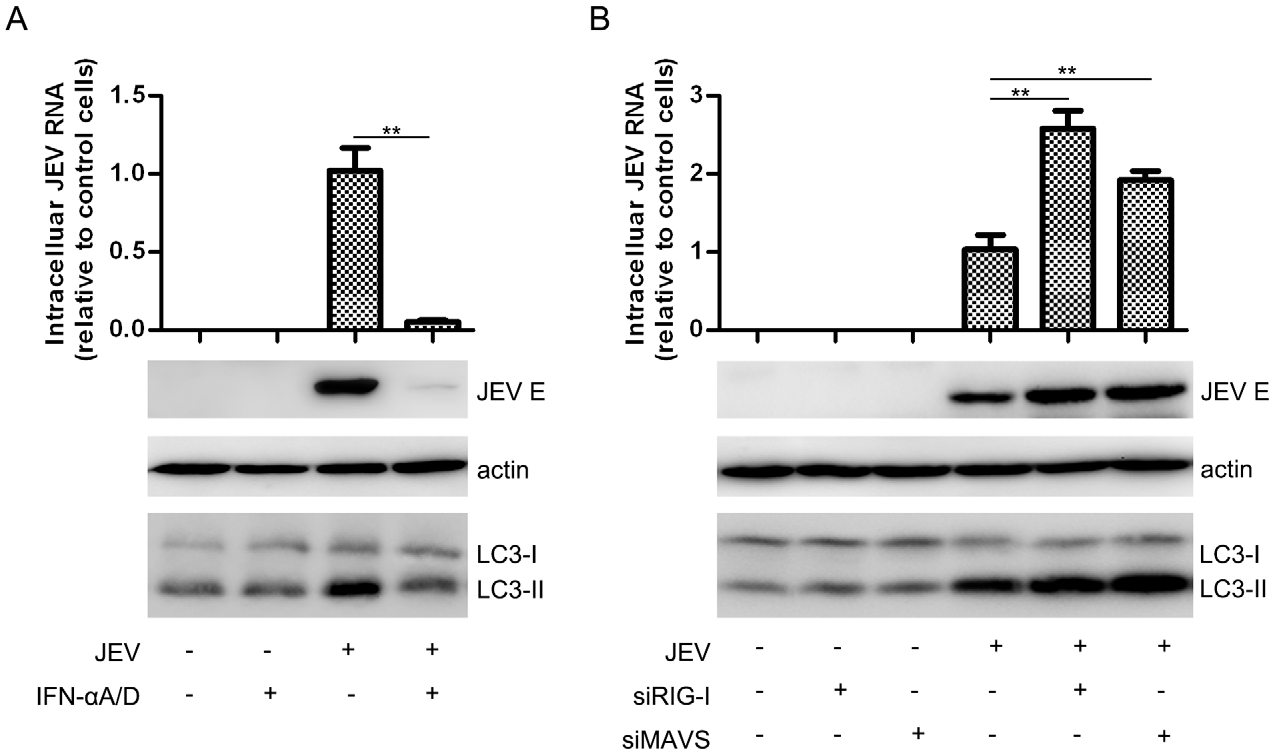
Autophagy-related type I IFN anti-viral pathway. (A) A549 cells were treated or mock-treated with IFN-αA/D (1,000 U/ml for 6 hours) and then infected or mock-infected with JEV (MOI = 0.1) for 48 hours. The cells were then harvested for JEV RNA and E protein analysis. (B) A549 cells were transfected with the indicated siRNA, and 48 hours later, the cells were infected or mock-infected with JEV (MOI = 0.1) for another 48 hours before being harvested for JEV RNA and E protein analysis. Three replicates were performed, and the results are presented as the means with SEM. The statistical significance of variations compared with the respective controls was calculated by t-test. **: *P*<0.01.

To further investigate the autophagy-related IFN signaling pathway, we used universal type I interferon, recombinant interferon-αA/D (IFN-αA/D, SIGMA). Cells were either pretreated or not pretreated with IFN-αA/D and were then infected or not infected with JEV at an MOI of 0.1. Virus replication and autophagy formation were then analyzed. The IFN-αA/D treatment nearly abolished JEV infection. However, the accumulation and induction of autophagy was not affected in the absence of infection (Fig. 6A). Furthermore, retinoic acid-inducible gene I (RIG-I, which is responsible for JEV-induced IFN β production and is essential for the detection of in vitro-transcribed dsRNAs [3]) and MAVS knock-down (the siRNA silencing effect was tested as shown in Fig. S5) A549 cells and wild-type cells were challenged with JEV infection. These knock-downs, intended to disrupt the IFN pathway, did not affect autophagosome formation in the absence of infection but did contribute to viral mRNA and protein expression and LC3-II accumulation during infection (Fig. 6B). These results suggest that autophagy is an upstream factor influencing the innate immune pathway in JEV infection.

**Figure 7:**
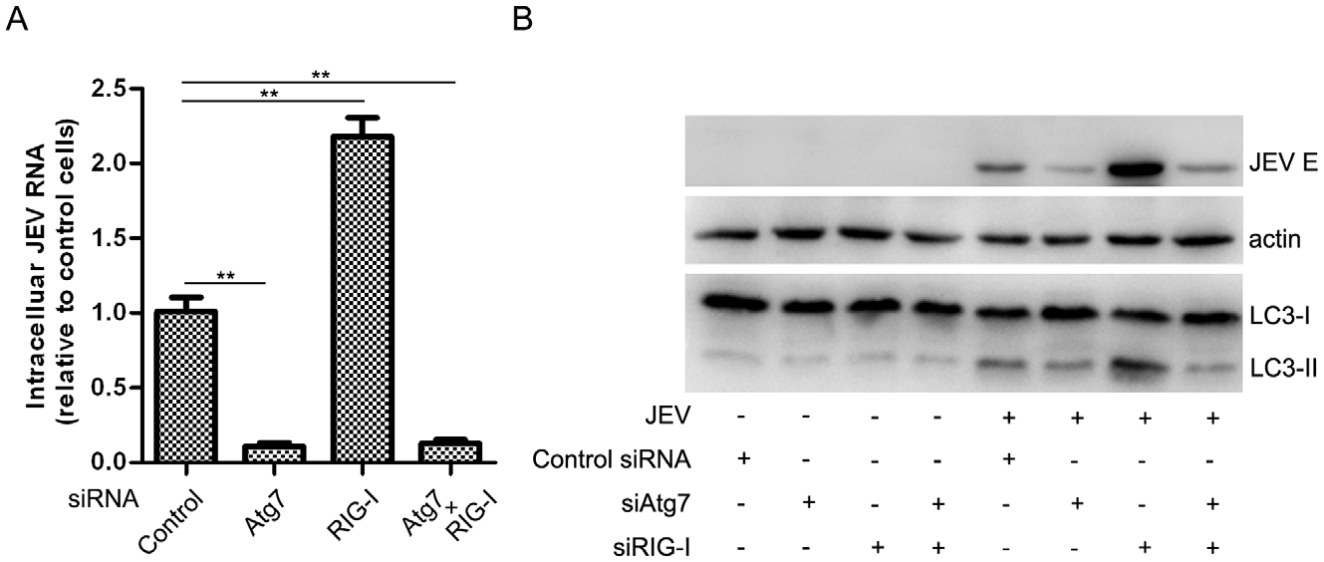
Autophagy is indispensable for JEV replication in the IFN-defective background. A549 cells were transfected with the indicated siRNA, and 48 hours later, the cells were infected or mock-infected with JEV (MOI = 0.1) for 48 hours before being harvested for JEV RNA (A) and E protein analysis (B). Three replicates were performed, and the results are presented as the means with SEM. The statistical significance compared with the control was calculated by t-test. **: *P*<0.01.

Although JEV activates autophagy to dampen MAVS-IRF3 activation to facilitate viral replication, as has been discussed, it would be interesting to test whether knock-down of the type I IFN signaling pathway could repair the viral replication defect in the case of autophagy dysfunction. As shown in Fig. 7, knock-down of Atg7 resulted in reductions in JEV replication at both the RNA and protein levels, a result consistent with those observed in both Atg5 and Beclin1 knock-downs (Fig. 3). We observed almost the same inhibition of efficiency of JEV replication in the presence of a RIG-I/Atg7 double knock-down, compared with an Atg7 knock-down alone. These findings demonstrated that autophagy is still required for JEV replication, even when the type I IFN signaling pathway is inhibited.

## Discussion

For numerous viruses, there is a connection between infection and autophagy. Some viruses have evolved mechanisms to hinder autophagy in infected cells to persist in their hosts. All three subfamilies of herpesviruses and lentiviruses encode viral proteins and/or induce cellular signals to inhibit autophagy [32], [33], but flaviviruses, such as DENV and HCV, have evolved mechanisms to benefit from autophagy in infected cells [7], [8]. However, how the accumulation of autophagosomes enhances JEV replication is not fully understood. In this study we defined another role for autophagy following JEV infection: the functional dampening of innate immune anti-viral responses. Silencing of autophagy-related genes led to the upregulation of type I IFN and cytokine expression, greater MAVS aggregation, and greater IRF3 dimerization and autophosphorylation.

Several studies have reported elevated RIG-I-like receptor (RLR) signaling mediated by autophagy following virus infection. Jounai et al. reported that the Atg5–Atg12 conjugate negatively regulates the type I IFN production pathway by direct association with RIG-I and MAVS through the caspase recruitment domains (CARDs) [27]. Moreover, Caspi et al. declared that the absence of autophagy results in ROS-dependent amplification of RLR signaling [34]. Recently, two reports about HCV demonstrated that the autophagy pathway was exploited to escape the innate immune anti-viral response [26], [35]; nevertheless, the exact physiological role that autophagy plays in innate immunity following JEV infection remains unclear. Recently, Hou et al. reported that MAVS converts to functional prion-like aggregates that potently activate IRF3 in the cytosol and propagate an anti-viral signaling cascade [31]. Earlier reports have indicated that when the cytosolic protein aggregated to form a poor proteasome substrate, autophagy then became the main clearance route. Under these circumstances, autophagy becomes more effective than the proteasome [36]. That is, the amplified IFN protein involved in MAVS degradation and signal feedback control mainly depends on the autophagy clearance route rather than the proteasome. Our data suggest that cells with disruptions in autophagy exhibit defects in mitochondrial metabolism and widespread aggregation of MAVS signal proteins, which should be degraded over time. The persistence of the amplified signal leads to a significant up-regulation in IFN expression. Besides the induction of IFN, autophagy is also required for the activation of NF-κB [37], therefore, it is plausible that dysfunctional autophagy led to the upregulation of IL6 and other cytokines. This is the first study to report that autophagy negatively regulates the innate immune response to facilitate JEV infection.

Although the exact mechanism involved in autophagy and innate immunity remains to be elucidated, further studies in this field are still warranted and needed, such as explorations of whether the Atg5-Atg12 complex directly affects MAVS aggregation. The interaction of the virus with the autophagy machinery involves multiple immunity pathways that have only just begun to be characterized.

In summary, JEV activates the cellular autophagy machinery to reduce the innate antiviral immune response and promote cell survival. Together, these effects allow for longer virus progeny production. To understand how these viral tactics affect pathogenesis and the viral lifecycle requires additional study. The elucidation of the signal network among autophagy, the immune response and JEV pathology will provide a basis for exploring other virally induced diseases.

## Materials and Methods

### Ethics Statement

This study was performed in strict accordance with the recommendations in the Guide for the Care and Use of Laboratory Animals according to the Hubei Administration Office of Laboratory Animals. The protocol was approved by the official Committee on the Ethics of Animal Experiments of Huazhong Agricultural University and Institutional Review Board of Wuhan Institute of Virology (Permit Number: WIVH25201201). All procedures were performed under isoflurane anesthesia, and all efforts were made to minimize suffering.

### Plasmids, Reagents and Antibodies

The mouse LC3B gene was cloned into the pEGFP-C3 or pDsRed-C1 vectors. Mouse LAMP1 was cloned into pEGFP-N1. DsRed-rab7 [38] (Addgene plasmid 12661) and mRFP-rab5 [39] (Addgene plasmid 14437) were described previously.

JEV E-specific antibodies for immunoblotting have been previously described [40]. The actin mAb (SC-1616-R) was purchased from Santa Cruz Biotechnology. In addition, p-IRF3 (4947), IRF3 (4302), caspase-9 (9504), caspase-3 (9662), and RIG-I (3743) antibodies were purchased from Cell Signaling Technology. LC3 mAb (L7543), 3-methyladenine (3-MA, M9281), thapsigargin (Tg, T9033), and Interferon-αA/D (14401) were purchased from Sigma-Aldrich. Lipofectamine™ 2000 Reagent (11668-019) and TRIzol (15596-026) were purchased from Invitrogen. MAVS mAb (ab59319) was purchased from Abcam. Atg5 (10181-2-AP) and Atg7 (10088-2-AP) antibodies were purchased from Proteintech. Cell viability was determined according to the manufacturer’s suggested protocol (Beyotime, C0038), and the details can be found in Protocol 1 in File S1.

### Cell Lines, Virus and Plaque Assays

N2a (purchased from ATCC), BHK21 and A549 cells were cultured in DMEM (Hyclone) or F-12K (Gibco) media, respectively, supplemented with 10% fetal bovine serum (Gibco) at 37°C in a 5% CO_2_ incubator. The propagation of the JEV strain SA14-14-2 was performed in BHK21 cells, and strain P3 was amplified in the brains of suckling mice [40]. Both were titered using a plaque assay with BHK21 cells. For the plaque assay, ten-fold dilutions of a virus stock were prepared and then inoculated onto susceptible BHK21 cell monolayers. After a 1-hour incubation period to allow the virus to attach to cells, the monolayers were covered with a nutrient medium (4% methyl cellulose, 4% FBS, 1% DMSO in DMEM) after removing the remaining virus stock. Three days later, the cells were stained with crystal violet and titered.

### Fluorescence and Confocal Microscopy

For the detection of autophagosomes and colocalization, N2a cells were grown to 80% confluence in a confocal dish (Wuxi NEST Biotechnology. Co., Ltd.) and were then transfected with the indicated plasmids. After 12 hours, they were treated or infected as planned. For starvation, the cells were incubated with Earle’s Balanced Salt Solution (EBSS, Gibco) for 2 hours. The cells were fixed (4% paraformaldehyde in PBS, 20 min) and permeabilized (0.3% Triton X-100 in PBS, 10 min), and the nuclei were stained with DAPI (Invitrogen). Cells containing three or more GFP-LC3 puncta, as observed under a fluorescence microscope (Olympus, IX71), were defined as autophagy-positive cells. Co-localization of early endosomes, late endosomes or lysosomes with autophagosomes were observed with a confocal ﬂuorescence microscope (ULTRAVIEW Vox).

### Animal Infections and Transmission Electron Microscopy

Mice used in this study were housed in positively ventilated microisolator cages that were kept in a constant temperature and humidity room in the Animal Facility of Huazhong Agricultural University. The animals received autoclaved food, water, bedding and filtered air. Four-week old naive BALB/c mice were first anesthetized with isoflurane and then injected with JEV P3 virus in the brain. The infected mice were monitored every 6 hours for 6 days until euthanized by cervical dislocation. Each brain was harvested and fixed with 2.5% glutaraldehyde in 0.1 M cacodylate buffer containing 4% sucrose. The tissue was then fixed in 1% OsO_4_ and dehydrated. Areas containing cells were block-mounted and thinly sliced. Finally, after staining with uranyl acetate and lead citrate, the ultrathin sections were examined with TEM (FEI TECNAI G2).

### SDS-PAGE, Native PAGE, SDD-AGE and Western Blotting

Cells were lysed in lysis buffer (50 mM Tris, 150 mM NaCl, 1 mM EDTA, 1% NP40, pH 7.4) containing a protease inhibitor cocktail (Roche). The protein concentration was determined using a BCA Protein Assay Kit (Beyotime, P0012). Equal amounts of protein were separated by SDS-PAGE and transferred onto a PVDF membrane (Millipore). After blocking with 5% nonfat milk in TBST (10 mM Tris, 150 mM NaCl, 0.1% Tween-20, pH 7.4), the membrane was incubated with specific primary antibodies overnight at 4°C. The blots were then incubated with HRP-conjugated secondary antibody (Proteintech) and visualized with a chemiluminescence system (Millipore, WBKL S0500). The SignalBoost™ Immunoreaction Enhancer Kit (Calbiochem, 407207) was used to resolve problems of low sensitivity. Native PAGE for IRF3 assays was performed as described previously [41], the details can be found in Protocol 2 in File S1. The crude mitochondria preparation and semi-denaturing detergent agarose gel electrophoresis (SDD-AGE) for assaying MAVS aggregation were performed as previously described [31], [42], the details can be found in Protocol 3 in File S1.

### Luciferase Assay

Cells co-transfected with IFN β reporter plasmid p125-luc (Stratagene) and pRL-TK (Promega) were challenged with JEV infection, then harvested at different times as indicated and analyzed for firefly and Renilla luciferase activities using a Dual-Luciferase Reporter Assay System (Promega, E1910) and a GloMax Multi Detection System (Promega).

### siRNA and RT-PCR

Cells were seeded evenly without antibiotics until 30–50% confluent. Transfection was performed with the indicated siRNA oligonucleotides using Lipofectamine™ 2000 according to the manufacturer’s instructions. For RNA interference, small interfering RNAs were purchased from GenePharma. We used the following oligonucleotides: mouse Atg5, sequence 5′-ACCGGAAACUCAUGGAAUA-3′ [43]; mouse Beclin1, sequence 5′-GAUCCUGGACCGGGUCACCTT-3′ [44]; human Atg7, sequence 5′-GCAUCAUCUUCGAAGUGAATT-3′ [8]. Other siRNAs, such as those for mouse Rab7 (EMU150241) and LAMP2 (EMU071371), were purchased from Sigma; human Atg5 (6354) was purchased from Cell Signaling Technology; human RIG-I (SI03019646) was purchased from Qiagen; human MAVS (SC-75755) was purchased from Santa Cruz. Relative changes in gene expression were calculated using the 2^−ΔΔCt^ method [45].

Total RNA was isolated using TRIzol reagent (Invitrogen) and reverse-transcribed using M-MLV Reverse Transcriptase (Invitrogen, AM2044) following the manufacturer’s directions. The resulting cDNA was then amplified using Fast SYBR Green Master Mix (ABI, 4385612) and a Quantitative Real Time-PCR system (ABI, Stepone). The sequences, suppliers and references for the primers are shown in the Table S1.

## Supporting Information

Figure S1 **Autphagosomes co-localized with early endosome markers.** N2a cells were co-transfected with GFP-LC3B and mRFP-Rab5 plasmid respectively, 12 hours after transfection cells were then challenged with JEV. 36 hours post-infection, the cells were fixed and the nuclei were stained. The cells were observed under a confocal ﬂuorescence microscope.
(DOC)

Figure S2 **Autphagosomes co-localized with late endosome markers.** N2a cells were co-transfected with GFP-LC3B and DsRed-Rab7 plasmid respectively, 12 hours after transfection cells were then challenged with JEV. 36 hours post-infection, the cells were fixed and the nuclei were stained. The cells were observed under a confocal ﬂuorescence microscope.
(DOC)

Figure S3 **The siRNA knock-down effect of Mouse Rab7 and LAMP2 was tested.** N2a cells were transfected with siRNA oligonucleotides against Mouse Rab7 and LAMP2, 72 hours later, the cells were harvested and lysed for RNA and protein analysis.
(DOC)

Figure S4 **The siRNA knock-down effect of Human Atg5 and Atg7** **was tested.** A549 cells were transfected with siRNA oligonucleotides against Human Atg5 and Atg7, 72 hours later, the cells were harvested and lysed for RNA and protein analysis.
(DOC)

Figure S5 **The siRNA knock-down effect of Human RIG-I and MAVS was tested.** A549 cells were transfected with siRNA oligonucleotides against Human RIG-I and MAVS, 72 hours later, the cells were harvested and lysed for protein analysis.
(DOC)

Table S1 **Real-time PCR primers.**
(DOC)

File S1 **Contains supporting protocols 1, 2 and 3.**
(DOC)
