# Correction: Japanese Encephalitis Virus Activates Autophagy as a Viral Immune Evasion Strategy

**DOI:** 10.1371/annotation/f7dcec2f-ed82-4a31-96c6-2953b421fd92

**Published:** 2013-05-13

**Authors:** Rui Jin, Wandi Zhu, Shengbo Cao, Rui Chen, Hui Jin, Yang Liu, Shaobo Wang, Wei Wang, Gengfu Xiao

The SDD-AGE experiment described in the article was carried out following the technical guidance from the publications by Halfmann et al. and Hou et al. below:

Halfmann R, Lindquist S (2008) Screening for amyloid aggregation by Semi-Denaturing Detergent-Agarose Gel Electrophoresis. J Vis Exp 17: 838.

Hou F, Sun L, Zheng H, Skaug B, Jiang QX, et al. (2011) MAVS forms functional prion-like aggregates to activate and propagate antiviral innate immune response. Cell 146: 448-461.

Although we have cited both references in the article (as references 42 and 31 respectively), the description of the methods in the File S1 contains overlap in text with these publications. The gel preparation in part 1 and transfer in part 4 of protocol S3 contain overlap in text with the publication by Halfmann et al., while the preparation of mitochondria in part2 and electrophoresis in part 3 contain overlap in text with the publication by Hou et al.

The authors acknowledge this mistake and earnestly apologize. 

