# Supplementary material for: Japanese Encephalitis Virus Activates Autophagy as a Viral Immune Evasion Strategy
Source: PLoS One. 2013 Jan 8;8(1):e52909. doi: 10.1371/journal.pone.0052909 (PMC3540057; doi:10.1371/journal.pone.0052909)
Supplement: Figure S2 — Autphagosomes co-localized with late endosome markers. N2a cells were co-transfected with GFP-LC3B and DsRed-Rab7 plasmid respectively, 12 hours after transfection cells were then challenged with JEV. 36 hours post-infection, the cells were fixed and the nuclei were stained. The cells were observed under a confocal ﬂuorescence microscope. (DOC) [file pone.0052909.s002.doc]

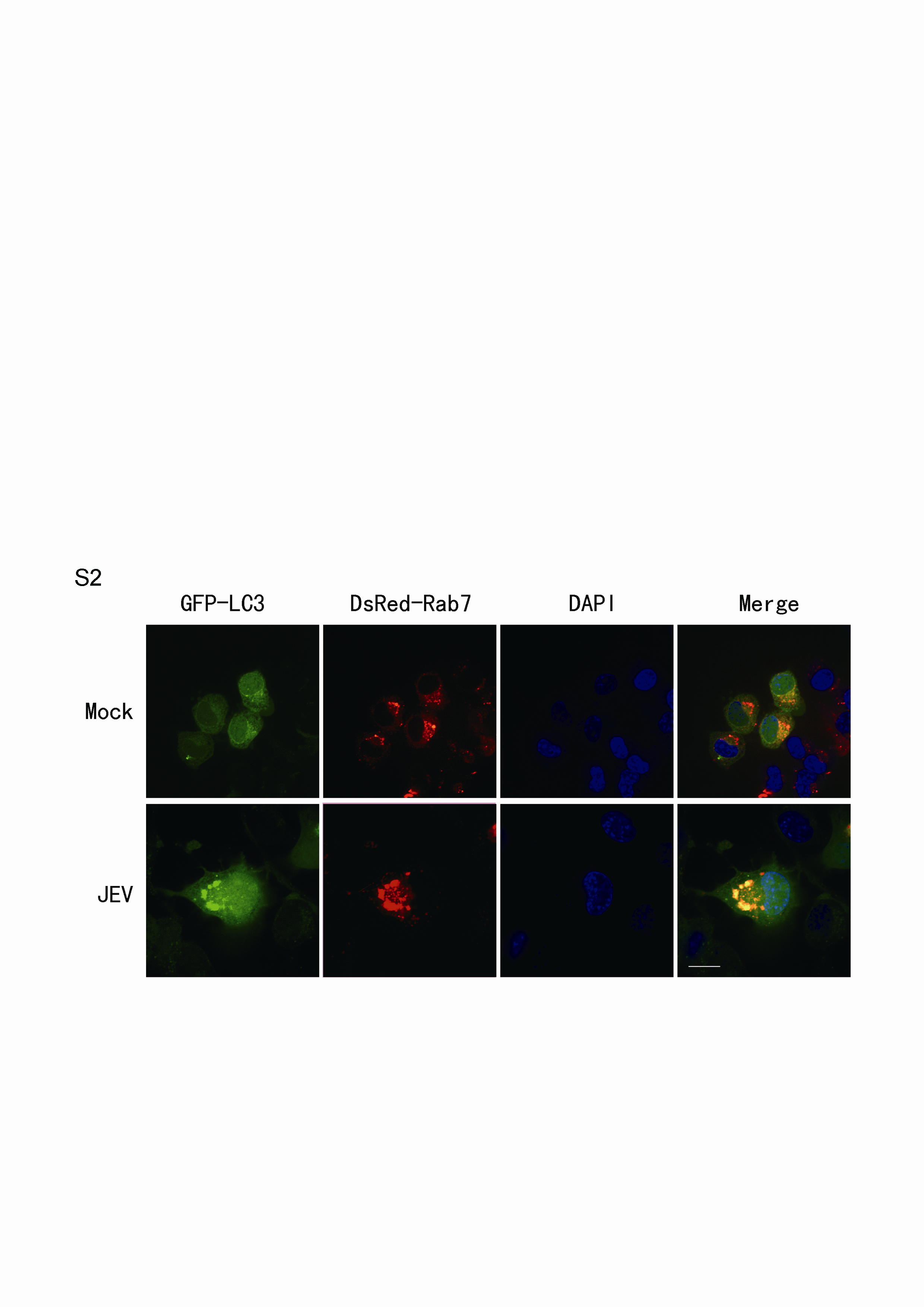


**Figure. S2 Autphagosomes co-localized with late endosome markers.** N2a cells were co-transfected with GFP-LC3B and DsRed-Rab7 plasmid respectively, 12 hours after transfection cells were then challenged with JEV. 36 hours post-infection, the cells were ﬁxed and the nuclei were stained. The cells were observed under a confocal ﬂuorescence microscope.
