# Supplementary material for: Japanese Encephalitis Virus Activates Autophagy as a Viral Immune Evasion Strategy
Source: PLoS One. 2013 Jan 8;8(1):e52909. doi: 10.1371/journal.pone.0052909 (PMC3540057; doi:10.1371/journal.pone.0052909)
Supplement: Figure S3 — The siRNA knock-down effect of Mouse Rab7 and LAMP2 was tested. N2a cells were transfected with siRNA oligonucleotides against Mouse Rab7 and LAMP2, 72 hours later, the cells were harvested and lysed for RNA and protein analysis. (DOC) [file pone.0052909.s003.doc]

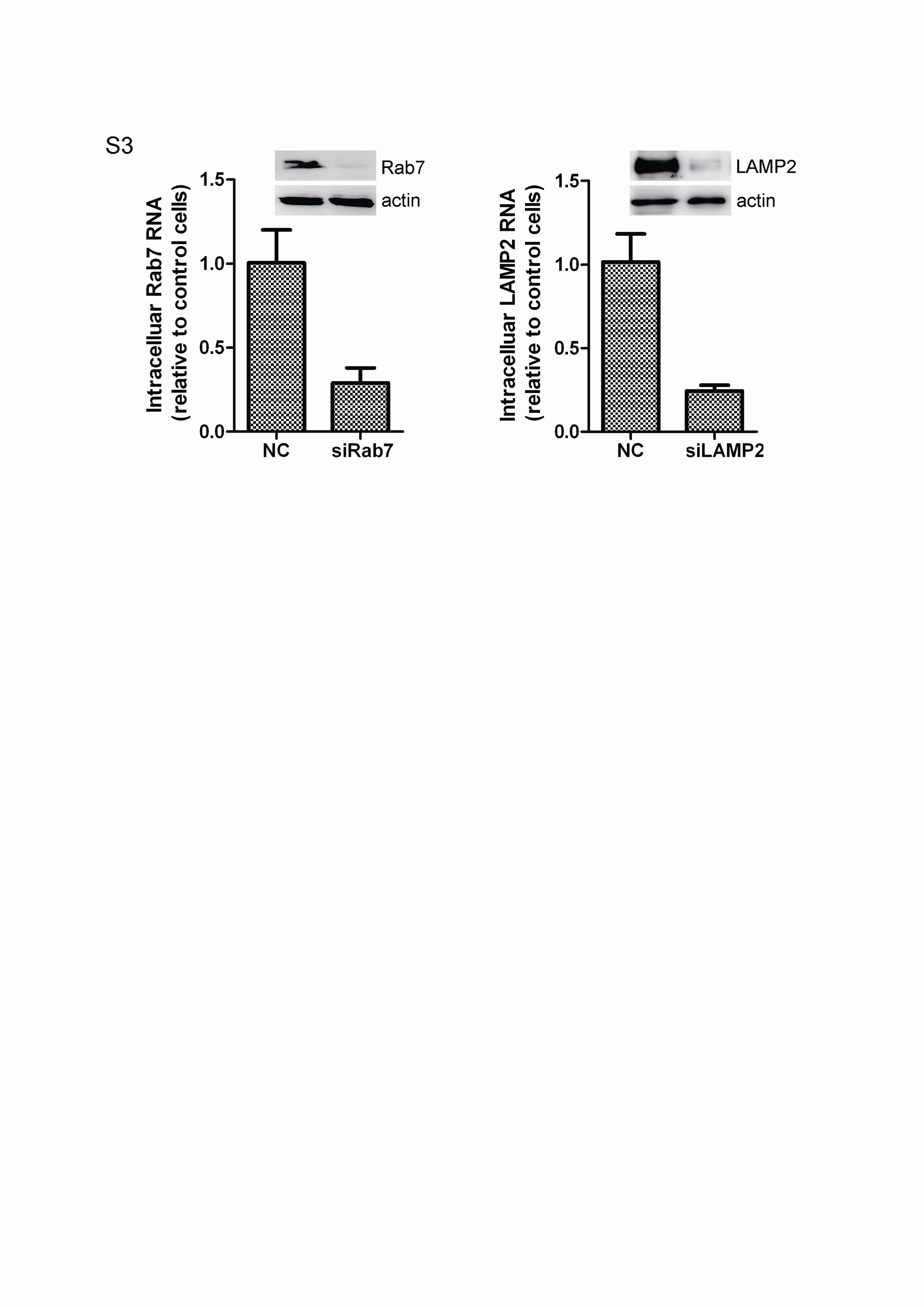


**Figure. S3 The siRNA knock-down effect of Mouse Rab7 and LAMP2 was tested.** N2a cells were transfected with siRNA oligonucleotides against Mouse Rab7 and LAMP2, 72 hours later, the cells were harvested and lysed for RNA and protein analysis.
