# Supplementary material for: Japanese Encephalitis Virus Activates Autophagy as a Viral Immune Evasion Strategy
Source: PLoS One. 2013 Jan 8;8(1):e52909. doi: 10.1371/journal.pone.0052909 (PMC3540057; doi:10.1371/journal.pone.0052909)
Supplement: Figure S4 — The siRNA knock-down effect of Human Atg5 and Atg7 was tested. A549 cells were transfected with siRNA oligonucleotides against Human Atg5 and Atg7, 72 hours later, the cells were harvested and lysed for RNA and protein analysis. (DOC) [file pone.0052909.s004.doc]

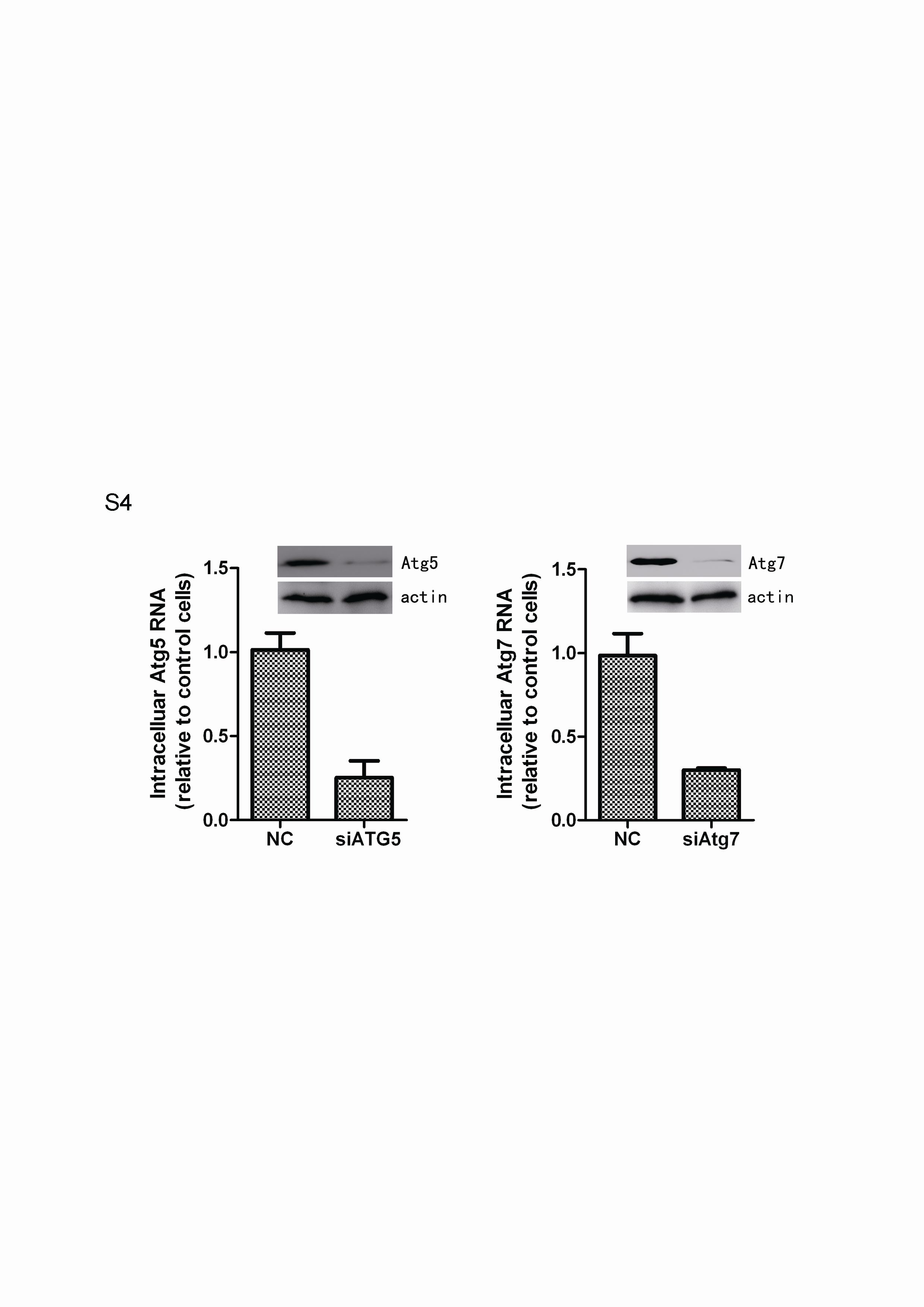


**Figure. S4 The siRNA knock-down effect of Human Atg5 and Atg7** **was tested.** A549 cells were transfected with siRNA oligonucleotides against Human Atg5 and Atg7, 72 hours later, the cells were harvested and lysed for RNA and protein analysis.
