# Supplementary material for: Japanese Encephalitis Virus Activates Autophagy as a Viral Immune Evasion Strategy
Source: PLoS One. 2013 Jan 8;8(1):e52909. doi: 10.1371/journal.pone.0052909 (PMC3540057; doi:10.1371/journal.pone.0052909)
Supplement: Figure S5 — The siRNA knock-down effect of Human RIG-I and MAVS was tested. A549 cells were transfected with siRNA oligonucleotides against Human RIG-I and MAVS, 72 hours later, the cells were harvested and lysed for protein analysis. (DOC) [file pone.0052909.s005.doc]

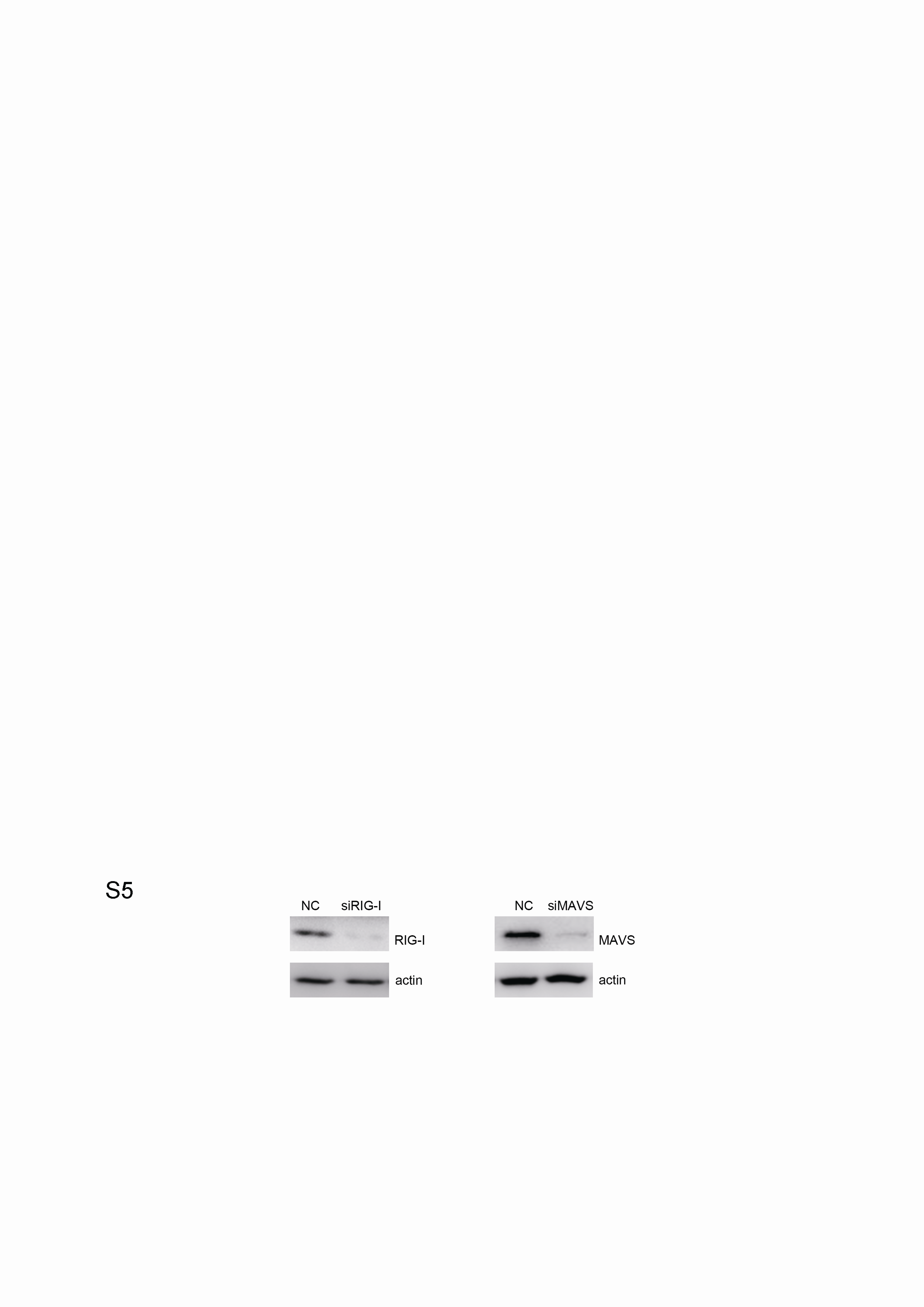


**Figure. S5 The siRNA knock-down effect of Human RIG-I and MAVS was tested.** A549 cells were transfected with siRNA oligonucleotides against Human RIG-I and MAVS, 72 hours later, the cells were harvested and lysed for protein analysis.
