# Supplementary material for: Japanese Encephalitis Virus Activates Autophagy as a Viral Immune Evasion Strategy
Source: PLoS One. 2013 Jan 8;8(1):e52909. doi: 10.1371/journal.pone.0052909 (PMC3540057; doi:10.1371/journal.pone.0052909)
Supplement: Table S1 — Real-time PCR primers. (DOC) [file pone.0052909.s006.doc]

**Table. S1** Real-time PCR primers

| Target cDNA | Forward primer | Reverse primer | Ref. |
| --- | --- | --- | --- |
| JEV | TACAACATGATGGGAAAAAGAGAGAAGAA | CTTGCTTTCCTGCTATGTCACGGAGGA | Cao S, et al. 2011. Virol J 8: 39. |
| Mus actin | CACTGCCGCATCCTCTTCCTCCC | CAATAGTCATGACCTGGCCGT | Cao S, et al. 2011. Virol J 8: 39. |
| Mus18S | TAGAGGGACAAGTGGCGTTC | CGCTGAGCCAGTCAGTGT | Tal MC, et al. 2009. PNAS 106: 2770-2775. |
| MusCOI fp | GCCCCAGATATAGCATTCCC | GTTCATCCTGTTCCTGCTCC | Tal MC, et al. 2009. PNAS 106: 2770-2775. |
| Hum Atg5 | GCAAGCCAGACAGGAAAAAG | GACCTTCAGTGGTCCGGTAA | Fei PW, et al. 2004. Cancer Cell 6: 597-609. |
| Hum Atg7 | AAAGCAGTTGGATGGGAAAAG | GCAGCAGACATTTGACAGACA |  |
| Hum GAPDH | ACCACCATGGAGAAGGCTGG | CTCAGTGTAGCCCAGGATGC | Takeshita F, et al. 2004. J Immunol 173: 2552-2561. |
| Mus LAMP2 | TGACATCGTGCTTTCCTACAA | GGGAGTTTGGTCTTCTTCACA |  |
| Mus Rab7 | ATCATCCTGGGGGACTCTG | CGCTCCTATTGTGGCTTTGT |  |
| Hum IL6 | CCCCCAGGAGAAGATTCCA | TCAATTCGTTCTGAAGAGGTGAG | Zhou Y, et al. 2010. PLoS ONE 5: e9224. |
| Hum IFN-β | CAGCAATTTTCAGTGTCAGAAGC | TCATCCTGTCCTTGAGGCAGT | Hayashi F, et al. 2003. Blood 102: 2660-2669. |
| Hum IP10 | TTCAAGGAGTACCTCTCTCTAG | CTGGATTCAGACATCTCTTCTC | Chakrabarti AK, et al. 2010. Virol J 7: 219. |
