# Supplementary material for: Japanese Encephalitis Virus Activates Autophagy as a Viral Immune Evasion Strategy
Source: PLoS One. 2013 Jan 8;8(1):e52909. doi: 10.1371/journal.pone.0052909 (PMC3540057; doi:10.1371/journal.pone.0052909)
Supplement: File S1 — Contains supporting protocols 1, 2 and 3. (DOC) [file pone.0052909.s007.doc]

**Supplementary Protocl**

**Protocol S1**

Cell viability assay

3×10^4^ cells/well were seeded in a 24-well ﬂat-bottomed plate, grown at 37 °C for 24 h, and then cells were transfected with siRNA oligonucleotides against Atg5 and Beclin1. Twenty-four hours later, the cells were infected at an MOI of 0.1 for 72 hours. After 50 µl WST-8 dye (Beyotime, C0038) was add to each well, cells were incubated at 37 °C for 2 hours and the absorbance was determined at 450 nm using a microplate reader.

**Protocol S2**

Native PAGE for IRF3 assays

Cells were lyzed in protein lysis buffer, then 10 µg of cell lysate in native sample buffer (62.5 mM Tris–Cl [pH 6.8], 15% glycerol, and 1% DOC) were separated by 7.5% PAGE (polyacrylamide:bis = 29:1) without SDS for 60 min at 25 mA. The electrophoresis buffer contained 25 mM Tris and 192 mM glycine (pH 8.4) with and without 1%DOC in the cathode and anode chambers, respectively

**Protocol S3**

The crude mitochondria preparation and semi-denaturing detergent agarose gel electrophoresis (SDD-AGE) for assaying MAVS aggregation

Part 1: Preparing the gel

1. Assemble the gel casting tray. Standard equipment for horizontal DNA electrophoresis can be used. For large numbers of samples, we prepare a 20 cm x 24 cm slab with up to four 50-well combs. Make sure the slab does not have scratches, as these can distort the blot image.
2. Create a 1.5% agarose solution (medium or high gel-strength, low EEO) in 1X TAE. The volume should be enough to completely submerge the comb teeth -- you will want to load as much sample as possible to maximize detection. Microwave the mixture until the agarose is completely dissolved.
3. Rapidly add SDS to 0.1% from a 10% stock. Swirl to mix. If some agarose solidifies during this step, redissolve using a hot plate and be careful to avoid boiling.
4. Pour the solution into the casting tray. Use a comb to rake out any bubbles, as they could later interfere with transfer.
5. After gel has set, remove combs and place the gel into the gel tank. Completely submerge the gel in 1X TAE containing 0.1% SDS.

Part 2: Preparing the mitochondria

Crude mitochondria and cytosolic extracts were prepared by differential entrifugation. In brief, cells were resuspended in Buffer A (10mM Tris-HCl [pH 7.5], 10mM KCl, 1.5mM MgCl2, 0.25M D-mannitol, and Roche EDTA-free protease inhibitor cocktail) and then lysed by repeated douncing. After removing the cell debris by centrifugation at 1000g for 5min, the supernatants were centrifuged at 10,000g for 10min at 4℃ to separate mitochondria and cytosolic extracts, the crude mitochondria is in the precipitation. The mitochondria can be further puriﬁed if needed.

Part 3: electrophoresis

Crude mitochondria were resuspended in sample buffer (0.5 X TBE, 10% glycerol, 2% SDS, and 0.0025% bromophenol blue) and loaded onto a vertical 1.5% agarose gel. After electrophoresis in the running buffer (TBE with 0.1% SDS) for 35min with a constant voltage of 100 V at 4℃.

Part 4: Transfer

1. Cut a piece of nitrocellulose to the same dimensions as the gel.
2. Cut 20 pieces of GB004 and 8 pieces of GB002 blotting paper, to the same dimensions as the gel. Cut an additional piece of GB004 to be used as a wick; make it about 20 centimeters wider than the gel.
3. Immerse the nitrocellulose, wick, and 4 pieces of GB002 in 1X TBS.
4. In a plastic container, assemble a stack of papers as follows: 20 pieces of dry GB004, then 4 pieces of dry GB002, then one piece of pre-wet GB002. Lay the nitrocellulose on top of this stack.
5. Rinse the gel on the casting tray briefly in water to remove excess running buffer. Then, carefully begin to slide the gel off the tray onto the stack. While sliding the gel off the tray, douse the membrane with TBS as necessary. The extra buffer helps prevent bubbles from becoming trapped under the gel. A transfer pipette works well for this purpose.
6. After the gel has been moved to the stack, check thoroughly for bubbles. If any are present, lift the edge of the gel and reapply buffer until the bubbles can be worked out.
7. Put the remaining three pre-wetted GB002 pieces on top of the gel. Ensure thorough contact between all layers by rolling a pipette firmly across the top of the stack.
8. Flank the transfer stack with two elevated trays containing TBS. Drape the pre-wet wick across the stack such that either end of the wick is submerged in TBS.
9. Cover the assembled transfer stack with an additional plastic tray bearing extra weight (e.g., a 500 ml bottle of water).
10. Allow the transfer to proceed for a minimum of three hours, or overnight.
11. After transfer, the membrane can be processed by standard Western blotting.
